# A protein-protein interaction map of the TNF-induced NF-κB signal transduction pathway

**DOI:** 10.1038/sdata.2018.289

**Published:** 2018-12-18

**Authors:** Emmy Van Quickelberghe, Delphine De Sutter, Geert van Loo, Sven Eyckerman, Kris Gevaert

**Affiliations:** 1 VIB Center for Medical Biotechnology, B-9000 Ghent, Belgium; 2 Department of Biomolecular Medicine, Ghent University, B-9000 Ghent, Belgium; 3 VIB Center for Inflammation Research, B-9052 Ghent, Belgium; 4 Department of Biomedical Molecular Biology, Ghent University, B-9052 Ghent, Belgium

**Keywords:** Biochemistry, Protein-protein interaction networks

## Abstract

Tumor Necrosis Factor (TNF) has a crucial role in inflammation, cell proliferation and cell death. Dysregulation of TNF receptor 1 (TNFR1)-induced Nuclear Factor-kappa B (NF-κB) signaling leads to chronic inflammation and is associated with several human inflammatory pathologies. Hence, TNF neutralization suppresses inflammation and attenuates inflammatory pathology. However, despite its beneficial effects, anti-TNF therapy suffers from efficacy issues and severe immune side effects. There is thus an urging need to identify novel targets for pharmaceutical intervention in the NF-κB signaling pathway. Here, we present a protein-protein interaction dataset of the TNFR1-induced signaling pathway. For this, we used Virotrap, a novel method for studying protein complexes without disrupting the cellular integrity, on 12 central proteins controlling NF-κB and cell death signaling, both under resting conditions as well as upon TNF stimulation. Our dataset reveals dynamic interactions in TNFR1-induced NF-κB signaling and identifies both known as well as novel interactors that may help to further unravel the molecular mechanisms steering TNF-induced inflammatory signaling and pathology.

## Background & Summary

Tumor Necrosis Factor (TNF) is a major inflammatory cytokine activating the transcription factor Nuclear Factor-kappa B (NF-κB), but TNF is also able to induce apoptosis and necroptosis^1,2^. Optimal regulation of TNF signaling is essential to maintain tissue homeostasis and prevent inflammatory pathology^3^. Most inflammatory effects of TNF are mediated through TNF Receptor 1 (TNFR1), and neutralization of TNFR1-NF-κB signaling, as seen with TNF antagonists, possesses beneficial effects in autoimmune and inflammatory syndromes^3–6^. However, TNF antagonism comes with efficacy issues and has overall long-term side effects^6,7^. Hence, new targets for pharmaceutical intervention in the NF-κB signaling pathway need to be identified^3,7^.

**Figure 1:**
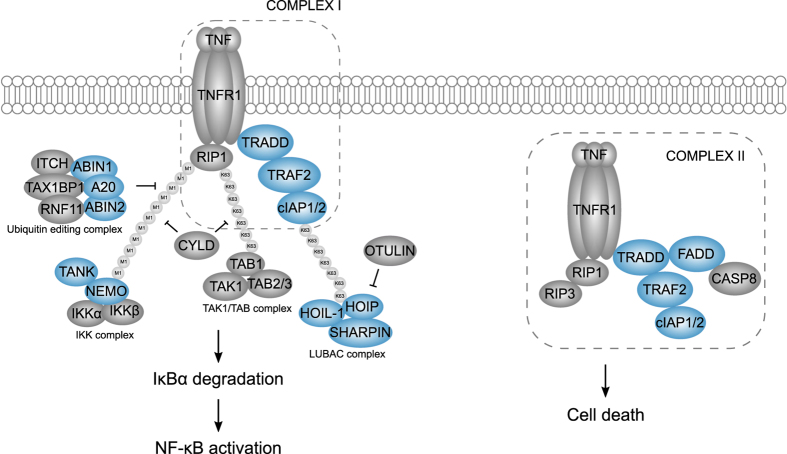
The TNFR1 signaling pathway leading to NF-κB activation or cell death. The TNF-bound TNFR1 is shown with the formation of complex I, leading to NF-κB activation on the left side, and formation of complex II, leading to cell death, on the right side. Ubiquitination modifications are shown, indicated as ‘M1’ for linear and ‘K63’ for lysine 63-linked ubiquitin chains. Proteins that were used as bait proteins in this study are indicated in blue.

Upon TNF binding, TNFR1 forms a trimer, thereby promoting the recruitment of the adaptor proteins TRADD (TNFR1-associated death domain protein) and RIP1 (Receptor-interacting serine/threonine-protein kinase 1). TRADD recruits TRAF (TNFR-associated factor) 2, which in turn recruits the ubiquitin ligases cIAP (cellular inhibitor of apoptosis) 1 and cIAP2. Together, these proteins form the membrane-bound receptor complex I^3,5^ (Fig. 1). The cIAP1/2 proteins promote auto-ubiquitination as well as ubiquitination of other downstream signaling proteins such as RIP1. K63-linked ubiquitination of cIAP1/2 leads to the recruitment of LUBAC (linear ubiquitination chain assembly complex), consisting of HOIL-1 (Heme-oxidized IRP2 ubiquitin ligase 1), HOIP (HOIL-1-interacting protein) and SHARPIN (Shank-associated RH domain-interacting protein), conjugating RIP1, NEMO (NF-κB essential modulator), TRADD and TNFR1 with linear polyubiquitin chains. These chains now serve as scaffolds for the recruitment and activation of the TAK1/TAB (TGF β-activated kinase 1/TAK1 binding protein) 2/3 and IKK (Inhibitor of kappa B (IκB) kinase) complexes^1,3,5^. Phosphorylation and activation of the IKK complex results in the phosphorylation of the NF-κB inhibitor protein IκBα, finally leading to its proteasomal degradation. NF-κB is now free to translocate to the nucleus and initiate transcription^2,8^. The adaptor protein TANK (TRAF family member-associated NF-κB activator) also functions in TNFR1-induced NF-κB signaling, although its precise role is not clear^9^ (Fig. 1).

NF-κB induces the transcription of anti-apoptotic genes, but also of a number of negative feedback regulators to fine-tune the overall signaling output^1,2^. CYLD, A20 and OTULIN are key negative regulators that attenuate TNFR1-mediated NF-κB signaling by counteracting the ubiquitination of signaling molecules^10^. A20 is thought to function as a ubiquitin-editing enzyme, in complex with a number of A20-binding proteins^10^.

Besides its importance in the activation of NF-κB, also another complex, cytosolic complex II, can assemble upon TNFR1 ligation, involving the dissociation of TRADD from complex I and the recruitment of FADD (Fas-associated death domain protein) and procaspase-8, leading to caspase-8 activation and apoptosis induction^3,5,8^ (Fig. 1).

This whole process of TNFR1-induced NF-κB signaling and cell death depends on protein-protein interactions (PPIs) and post-translational modifications. Hence, we hypothesized that mass spectrometry (MS)-based methods are better suited than genetic approaches^11,12^ to study the dynamics of these particular molecular interactions. PPI maps of the NF-κB pathway using tandem-affinity purification MS (TAP-MS) have been reported^13,14^ however, this and in fact, most AP-MS-based approaches to study PPIs require homogenization of cells expressing a bait protein of interest. With homogenization leading to loss of cellular structures, this may also reduce (local) protein concentrations and may affect the integrity of protein complexes. Furthermore, AP-MS typically requires several purification and washing steps, which may lead to the loss of (weaker) interactions. We recently developed a new method for studying protein complexes without interfering with cellular integrity: Virotrap^15,16^. By trapping and purifying protein complexes in virus-like particles, we avoid cell lysis and preserve intact complexes during the whole purification process. We showed that Virotrap captures significant parts of known interactomes, reveals new interactions, is able to detect weaker associations and is thus complementary to other PPI methods^15,17^.

Here, we present a protein-protein interaction network of the TNFR1-induced NF-κB and cell death pathway, using TRADD, FADD, TRAF2, cIAP1, NEMO, TANK, HOIL-1, HOIP, SHARPIN, A20, ABIN (A20-binding inhibitor of NF-κB activation) 1 and ABIN2 as baits in Virotrap (indicated in blue in Fig. 1). Virotrap has been applied both under resting conditions as well as after TNF stimulation, revealing dynamic interactions in TNFR1-induced NF-κB signaling. Our interaction map identifies both known protein partners as well as novel ones, and provides a valuable resource for further studying the molecular mechanisms steering inflammatory signaling processes and cell death.

## Methods

### Plasmids

The Gag-bait fusion constructs were generated as previously described^15,16^. The DNAs for genes of interest and the genes for control experiments were transferred from the human ORFeome 5.1 collection (http://horfdb.dfci.harvard.edu) into the pMET7-GAG-HRAS plasmid (Addgene plasmid #80604) by classic cloning with restriction enzymes. A detailed overview of the Gag-bait fusion plasmids used for the Virotrap experiment can be found in the Figshare repository (Plasmids, Data Citation 2). The pMD2.G (expressing VSV-G) and pcDNA3-FLAG-VSV-G plasmids are available at Addgene (#12259 and #80606). The TNFR plasmid (pSV25S-hTNFR55) was a gift from Rudi Beyaert (VIB Center for Inflammation Research, B-9052 Ghent, Belgium). The pcDNA2-HA-AMOT plasmid for the validation of the anti-AMOT antibody is available at Addgene (#32821). Plasmid pMET7-FLAG-NEMO for the validation of anti-NEMO antibody was constructed by transferring the DNA for NEMO from the human ORFeome 5.1 collection into a GATEWAY-compatible pMET7 expression vector with an amino-terminal FLAG-tag fused in-frame.

### Protein complex purification by Virotrap

**Figure 2:**
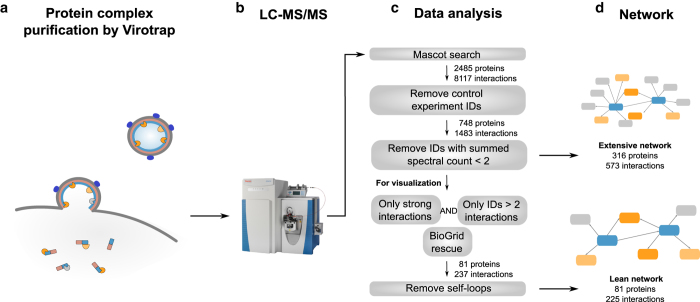
Workflow to generate the protein-protein interaction dataset. (**a**) Gag-bait fusion proteins were expressed in HEK293T cells and virus-like particles, trapping the protein complexes, were purified and prepared for subsequent LC-MS/MS analysis (**b**). Three biological replicates for every bait protein were performed, both for experiments without and with TNF treatment. (**c**) The MS/MS-data were searched with MASCOT and resulted in 2485 identified proteins. All proteins identified in a control experiment were removed, resulting in 748 remaining proteins. Interacting proteins identified with a single PSM were removed, resulting in an extensive network (**d**) with 316 proteins and 573 interactions. For network visualization, the dataset was further filtered (**c**). Only strong interactions (based on spectral counts) and interacting proteins with more than three interactions were withheld, unless the interacting protein was a known interactor in BioGRID. (**d**) The lean network consists of 81 proteins and 225 interactions.

The Virotrap protocol (Fig. 2a) is described in detail elsewhere^15^. HEK293T cells were cultured at 37°C and 8% CO_2_ in DMEM (Thermo Fisher Scientific, cat. no. 31966021), supplemented with 10% fetal bovine serum (Thermo Fisher Scientific, cat. no. 10270106) and 25 units/ml penicillin, 25 μg/ml streptomycin (Thermo Fisher Scientific, cat. no. 15070063). Cell cultures were kept at low passage (<10) and regularly tested for mycoplasma contamination. For every experiment, two 175 cm^2^ falcons with 23.3 × 10^6^ cells were seeded the day before transfection. Cells were transfected using polyethylenemine (PEI, Polysciences, cat. no. 23966) with a DNA mixture containing 17.5 μg bait plasmid (pMET7-GAG-bait), 12.5 μg pSVsport plasmid, 3.33 μg pcDNA3-FLAG-VSV-G plasmid and 1.67 μg pMD2.G plasmid. For Virotrap experiments with TNF treatment, 2.3 μg of pSVsport plasmid was replaced by pSV25S-hTNFR55 plasmid. After 6–8 h, the medium was refreshed with 18 ml supplemented DMEM per falcon. For Virotrap experiments with TNF treatment, cells were treated with 300 U/ml human TNF (made in-house) for 16 h.

40 h post-transfection, cells were visually inspected before purification of virus-like particles. Cell culture supernatant of two 175 cm^2^ falcons were combined (36 ml in total) and centrifuged at 1,500 g for 3 min at room temperature. The centrifuged supernatant was filtered through a 0.45 μm filter (Merck Millipore, cat. no. SLGV033RS). For every experiment, 100 μl MyOne Streptavidin T1 beads in suspension (10 mg/ml, Thermo Fisher Scientific, cat. no. 65601) were washed with 300 μl wash buffer containing 20 mM Tris-HCl (MP Biomedicals, cat. no. 02194558) pH 7.5 and 150 mM NaCl (Sigma, cat. no. S6191) and pre-loaded with 10 μl biotinylated anti-FLAG antibody (BioM2, Sigma, cat. no. F9291) in 500 μl wash buffer for 10 min at room temperature. Particles were allowed to bind for 2 h at room temperature by end-over-end rotation and the total supernatant was processed in three consecutive binding steps of 12 ml medium each. Bead-particle complexes were washed once with 1 ml and once with 500 μl wash buffer and eluted with 200 μg/ml FLAG peptide (Sigma, cat. no. F3290) in 100 μl wash buffer for 30 min at 37 °C. Particles were lysed by addition of SDS (sodium dodecyl sulfate, Merck Millipore, cat. no. 817034) to a final concentration of 0.1% for 5 min, followed by removal of SDS using HiPPR detergent removal columns (Thermo Fisher Scientific, cat. no. 88306) per manufacturer’s instructions. Proteins were heated at 95 °C for 5 min, cooled on ice at room temperature for 5 min and digested overnight with 1 μg sequence-grade trypsin (Promega, cat. no. V5111). Peptide mixtures were acidified with trifluoroacetic acid (TFA, Biosolve, cat. no. 202341) to a final concentration of 0.1% for LC-MS/MS analysis.

### LC-MS/MS analysis

2.5 μl of the obtained peptide mixtures was introduced into an LC-MS/MS system through an Ultimate 3000 RSLC nano LC (Thermo Fisher Scientific) in-line connected to a Q Exactive mass spectrometer (Thermo Fisher Scientific) (Fig. 2b). The sample mixture was first loaded on a trapping column (made in-house, 100 μm internal diameter (I.D.) × 20 mm, 5 μm beads C18 Reprosil-HD, Dr. Maisch). After flushing from the trapping column, the sample was loaded on an analytical column (made in-house, 75 μm I.D. × 150 mm, 5 μm beads C18 Reprosil-HD, Dr. Maisch) packed in the needle (PicoFrit SELF/P PicoTip emitter, PF360-75-15-N-5, New Objective). Peptides were loaded with loading solvent (0.1% TFA in water/acetonitrile (ACN, Biosolve, cat. no. 012007), 98/2 (v/v)) and separated with a linear gradient from 98% solvent A’ (0.1% formic acid (FA, Biosolve, cat. no. 069141) in water) to 40% solvent Bʼ (0.1% FA in water/ACN, 20/80 (v/v)) in 30 min at a flow rate of 300 nL/min. This was followed by a 5 min wash reaching 99% solvent B’. Two trapping columns and two analytical columns were configured in tandem LC mode, with switching between two flow paths – an analysis flow path and a regeneration flow path – allowing for column washing and re-equilibration off-line; thus, while one column is re-equilibrated, the system separates a sample on the other column.

The mass spectrometer was operated in data-dependent, positive ionization mode, automatically switching between MS and MS/MS acquisition for the 10 most abundant peaks in a given MS spectrum. The source voltage was 3.5 kV and the capillary temperature was set to 275 °C. One MS1 scan (m/z 400–2,000, AGC target 3 × 10^6^ ions, maximum ion injection time 80 ms) acquired at a resolution of 70,000 (at m/z 200) was followed by up to 10 tandem MS scans (resolution 17,500 at m/z 200) of the most intense ions fulfilling predefined selection criteria (AGC target 5 × 10^4^ ions, maximum ion injection time 60 ms, isolation window 2 Da, fixed first mass m/z 140, spectrum data type: centroid, underfill ratio 2%, intensity threshold 1.7 × 10^4^, exclusion of unassigned, 1, 5–8, >8 charged precursors, peptide match preferred, exclude isotopes on, dynamic exclusion time 20 s). The HCD collision energy was set to 25% Normalized Collision Energy (NCE) and the polydimethylcyclosiloxane background ion at 445.120025 Da was used for internal calibration (lock mass).

### Data analysis

The generated MS/MS peak lists were searched with the Mascot algorithm using the Mascot Daemon interface (version 2.5.1, www.matrixscience.com) at 99% confidence against the human (*Homo sapiens*) protein sequences available in the Swiss-Prot database (release of February 2015, containing 20,198 human entries), complemented with Gag, EGFP, eDHFR, VSV-G and FLAG-VSV-G protein sequences (Fig. 2c). Acetylation of protein N-termini, pyroglutamate formation of N-terminal glutamine and methionine oxidation to methionine-sulfoxide were set as variable modifications. Mass tolerance on precursor ions was set to 10 ppm and on fragment ions to 20 mmu. Trypsin was set as enzyme allowing for one missed cleavage. The peptide charge was set to 1+, 2+, 3+, and instrument setting was put to ESI-QUAD. False discovery rates (FDR) were obtained by repeating the searches against decoy database in which the sequences have been reversed and by retaining only the peptide to spectrum matches (PSMs) with the highest score in standard or reverse search. FDRs were then calculated by dividing the number of PSMs against the reverse database by the number of PSMs against both databases. The mass spectrometry proteomics data have been deposited to the ProteomeXchange Consortium via the PRIDE partner repository (Data Citation 1). An overview of all the LC-MS/MS runs with the number of spectra, PSMs, identified peptide sequences and identified proteins, as well as PSM FDRs can be found in the Figshare repository (Experiment Overview, Data Citation 2).

In total, 2485 proteins were identified (hits against the reversed database were omitted) and the spectral counts were extracted for every experiment (Spectral Counts, Data Citation 2). We applied a previously published stringent filtering approach (Fig. 2c)^15,18^ by removing every protein from the candidate interacting protein lists that was identified in at least one of the control experiments, which were processed and run under exactly the same conditions as the experiments with the proteins of interest. These control experiments consist of 15 Virotrap experiments with bait proteins that are not known to be involved in TNFR1-induced signaling (Experiment Overview, Data Citation 2). Further, when a protein was identified by only one peptide in a control experiment (as can be verified in Spectral Counts, Data Citation 2), the protein was removed from the list of possible interactors. This resulted in 748 remaining identified proteins (Fig. 2; Interactions filtered by controls, Data Citation 2). Next, the spectral counts for every withheld protein in a group of replicate experiments were summed. We further filtered the list of possible interactors by removing those that were only identified by a single PSM (i.e., the one-hit wonders) even though this PSM passed the stringent Mascot filtering criteria (see above). This filtering step resulted in an extensive network of 316 proteins and 573 interactions (Extensive network and Extensive network cytoscape file, Data Citation 2). The protein interactions from this extensive network have been submitted to the IMEx (http://www.imexconsortium.org) consortium through IntAct (Data Citation 3). Based on the summed spectral count, the remaining interactions were further assigned as weak interactions (spectral count = 2) or strong interactions (spectral count ≥ 3) for both the condition without treatment and the TNF treatment condition. For comparison with BioGRID, interaction proteins in the BioGRID database^19^ (Version 3.4.158, www.thebiogrid.org) for the 12 bait proteins were collected.

**Figure 3:**
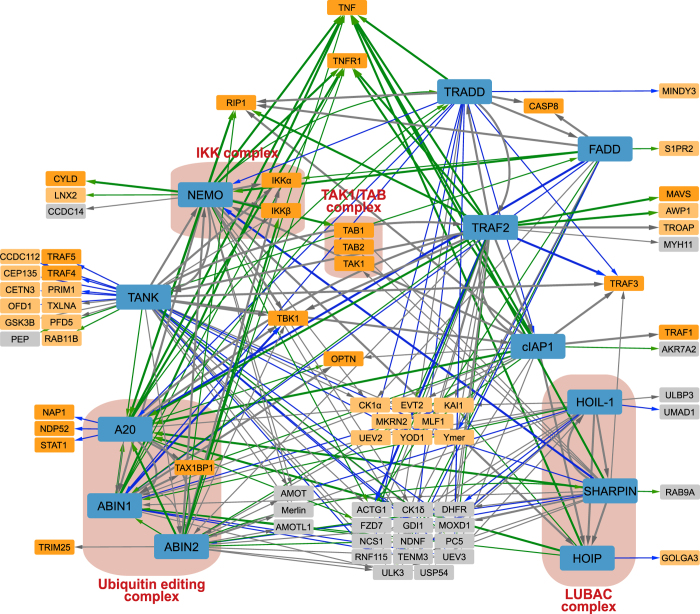
Lean network visualization. The bait proteins are represented by blue nodes. The nodes of interacting proteins are represented smaller and in intense orange when they have a well-known function in NF-κB and/or cell death signaling, in light orange when they can be linked to NF-κB and/or cell death signaling, and in grey when no function or link with NF-κB and/or cell death signaling is known so far. Interactions (edges) are represented by arrows that point from the bait protein to the identified interacting protein. Interactions that are independent of TNF treatment are shown in grey, interactions that appear or disappear upon TNF treatment are shown in green and blue respectively. Known interactions in the BioGRID database are represented by thicker lines (edges). Well-known protein complexes are shown in red boxes.

For better network visualization, we further filtered the extensive network. Only interactions that were assigned as strong interactions, and only interacting proteins identified with at least three interactions in the dataset (=nodes with at least three edges in the network) were withheld. However, to prevent false negative interactions, we kept interacting proteins that are known interacting partners for one of the baits in BioGRID. In this way, 44 proteins and 51 interactions that would otherwise be omitted, were rescued. Bait proteins interacting with themselves (self-loops in the network) were removed (Fig. 2c). This resulted in a lean network with 81 proteins (nodes) and 225 interactions (edges) (Fig. 3; Lean network and Lean network cytoscape file, Data Citation 2). Visualization of the networks was done with the Cytoscape 3 software (Version 3.2.1, www.cytoscape.org). The Cytoscape files of both the extensive network and the lean network are available for interactive network analysis (Extensive network cytoscape file and Lean network cytoscape file, Data Citation 2).

### Co-immunopurification and Western blot

Anti-AMOT (C-18, sc-82491, Santa Cruz Biotechnology) antibody was used for co-immunoprecipitation and Western blotting. IgG1 isotype control (sc-2028, Santa Cruz Biotechnology) antibodies was used for co-immunoprecipitation experiments and anti-NEMO (B-3, sc-8032, Santa Cruz Biotechnology) was used for Western blotting.

For every co-immunopurification experiment, 2 × 10^7^ HEK293T cells were seeded. After 48 h, cells were washed twice with 2 ml PBS (Invitrogen) and lysed in 500 μl lysis buffer, containing 10 mM Tris-HCl pH 8, 150 mM NaCl, 1% NP-40, 10% glycerol, 5 μM ZnCl_2_, 1 mM sodium orthovanadate, 20 mM β-glycerophosphate, 1 mM NaF and complete protease inhibitor cocktail (Roche). Lysates were centrifuged 15 min at 4 °C at 20 000×g. For every experiment, 10 μl MyOne ProteinG beads in suspension (10 mg/ml, Thermo Fisher Scientific, cat. no. 1004D) were washed with 300 μl wash buffer and preloaded with 1 μg anti-AMOT or IgG isotype control antibody in 200 μl wash buffer for 30 min at room temperature. Protein complexes were allowed to bind for 2 h by end-over-end rotation at 4 °C. The beads were washed twice with 200 μl lysis buffer, followed by elution in 30 μl SDS-PAGE (polyacrylamide gel electrophoresis) loading buffer (XT Sample Buffer and XT Reducing Agent, BioRad Laboratories) at 95 °C for 5 min.

For antibody validation, 2 × 10^7^ HEK293T cells were seeded the day before transfection. Cells were transfected using PEI with 7.5 μg pcDNA3-HA-AMOT plasmid or the combination of 7.5 μg pcDNA3-HA-AMOT and 7.5 μg pMET7-FLAG-NEMO plasmid. After 6–8 h, the medium was refreshed. 40 h post-transfection, cells were washed twice with 2 ml PBS (Invitrogen) and lysed in 500 μl lysis buffer. Proteins were denatured in 30 μl SDS-PAGE loading buffer and heated at 95 °C for 5 min.

**Figure 4:**
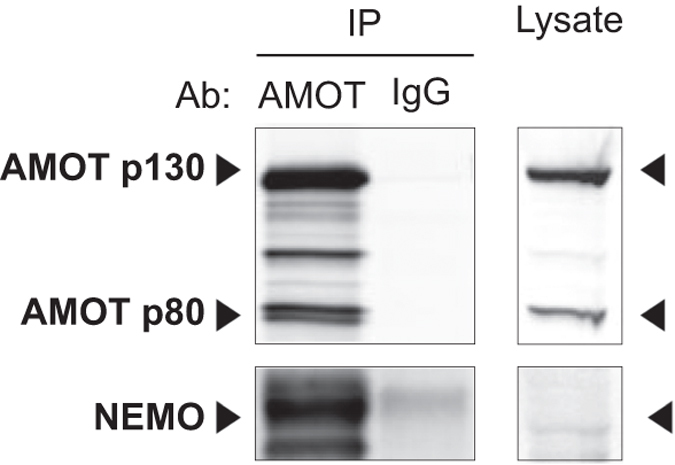
Co-immunoprecipitation analysis on endogenous expression level. HEK293T cell lysates were incubated with Protein G beads loaded with either anti-AMOT or IgG isotype control antibody. After 2 h of incubation, the beads were washed twice in lysis buffer and protein complexes were eluted from the beads in SDS-PAGE loading buffer. Immunoblots were sequentially incubated with anti-AMOT and anti-NEMO antibody. Immunoprecipitation (IP) of AMOT (both p80 and p130 isoforms) and co-immunoprecipitation of NEMO is clearly visible. One experiment of three biological replicate experiments is shown. Full Western blots of all three replicate experiment and antibody validation can be found in the Figshare repository (Uncropped Western blots, Data Citation 2).

Protein mixtures were separated by SDS-PAGE on a Criterion XT 4–12% Bis-Tris gel (Biorad Laboratories). Proteins were transferred to a PVDF membrane (Merck) after which blocking of the membrane was done in Odyssey Blocking Buffer (LI-COR). Immunoblots were incubated overnight at 4 °C with primary antibodies against AMOT and NEMO in Odyssey Blocking Buffer supplemented with 0.1% Tween-20 (v/v). Blots were washed four times with TBST (TBS supplemented with 0.1% Tween-20 (v/v)), incubated with fluorescent-labeled secondary antibodies (LI-COR) in Odyssey Blocking Buffer (LI-COR) supplemented with 0.1% Tween-20 (v/v) for 1 h at room temperature. After four washes with TBST, immunoblots were imaged using the Odyssey Infrared Imaging System (LI-COR). Uncropped scans of the Western blot in Fig. 4, as well as uncropped scans of the Western blots of replicate experiments and uncropped scans of the Western blots for antibody validation are available in the Figshare repository (Uncropped Western blots, Data Citation 2).

## Data Records

By applying Virotrap, we acquired a dynamic protein-protein interaction map involving multiple proteins that play a central role in TNFR1-induced NF-κB signaling and cell death. Fusion proteins of the HIV-1 Gag protein and the bait protein of interest are expressed in HEK293T cells, protein complexes are trapped in virus-like particles, followed by purification of the particles and identification of their protein content by LC-MS/MS (Fig. 2). All experiments were performed in three biological replicates, both without and with TNF treatment. The mass spectrometry proteomics data have been deposited to the ProteomeXchange Consortium via the PRIDE partner repository (Data Citation 1).

Processed data are available in the Figshare repository (Data Citation 2) including:

- a table with all plasmids used for Virotrap experiments (Plasmids),
- a table giving an overview of all Virotrap LC-MS/MS runs (Experiment overview),
- a table with spectral counts for every identified protein for every experiment (Spectral Counts),
- a table with all interactions after removing identifications from control experiments (Interactions filtered by controls),
- a table with the interactions in the extensive network (Extensive network),
- a table with the interactions in the lean network (Lean network),
- a table with references linking novel interacting proteins to NF-κB and/or cell death signaling (Novel interactors with link),
- a table showing the overlap with BioGRID after each filtering step (Overview filtering),
- a table with the results of the iMixPro AP-MS data for the A20 protein (A20 iMixPro AP-MS result),
- the Cytoscape files of the extensive network and the lean network,
- A figure showing uncropped Western blots of the co-immunoprecipitation experiments (Uncropped Western blots),
- A figure showing ratio histograms for quantified proteins and peptides for A20 as bait protein in the iMixPro AP-MS approach (iMixPro ratio histograms).

The protein interactions from the extensive network have been submitted to the IMEx (http://www.imexconsortium.org) consortium through IntAct (Data Citation 3).

## Technical Validation

The data were obtained from three independent biological replicates. An overview of the number of spectra, peptide to spectrum matches (PSM), identified peptide sequences and identified proteins, as well as PSM false discovery rates (FDR) can be found in the Figshare repository (Experiment overview, Data Citation 2).

In total, 2485 proteins were identified, corresponding to 8117 interactions. Of those 8117 interactions, 107 interactions were present in the BioGRID database for the 12 bait proteins in our study. To filter out possible false positive interactions, we removed every candidate interacting protein that was identified in at least one of the control experiments. These control experiments consist of 15 Virotrap experiments with bait proteins that are not known to be involved in TNFR1-induced signaling (Experiment Overview, Data Citation 2). These samples were processed and run under exactly the same conditions as the experiments with the proteins of interest. This resulted in a network of 748 proteins and 1483 interactions. Of note, this first filtering step did not remove any of the 107 interactions that were present in the BioGRID database for the 12 proteins (Overview filtering, Data Citation 2). Next, we also removed every candidate interacting protein that was identified with a single PSM and the resulting network contained 316 proteins and 573 interactions. However, likely because of their low abundance, we hereby lose 21 of the 107 interactions (i.e., 19.6%) present in the BioGRID database (Overview filtering, Data Citation 2). We seem thus to lose true interactions with this filtering step however, we hypothesize that one may not simply rely on a single PSM obtained over three biological replicate experiments to consider a protein as a true hit. The resulting network, which we call the extensive network (Extensive network, Data Citation 2 and Data Citation 3), thus contains 316 proteins and 573 interactions, of which 86 interactions (15%) are listed in the BioGRID database.

Simply for network visualization purposes, we further filtered this extensive network and withheld only strong interactions (summed spectral count > 2), and only interacting proteins identified with at least three interactions in the dataset, unless the interacting protein was a known interacting partner for one of the baits in BioGRID (to prevent false negative interactions, Fig. 2). This resulted in a network of 81 proteins and 225 interactions, which we call the lean network (Lean network, Data Citation 2).

The lean network (Fig. 3) contains many edges connecting the nodes as most of the bait proteins are identified as prey proteins of other bait proteins. Indeed, the 12 bait proteins in our dataset are closely interwoven, indicating (a) stable protein complex(es). This lean network contains 75 interactions known in BioGRID, including the rescued interactions during the filtering. We lose another 11 known interactions in BioGRID since we removed self-loops in the network for visualization (Overview filtering, Data Citation 2). The 150 novel interactions correspond to 72 proteins, of which 24 proteins have a well-known function in NF-κB signaling. Of the remaining 48 identified proteins, 23 proteins can be linked to NF-κB and/or cell death signaling, a strong indication that these novel interactions are true positive interactions (Novel interactors with link, Data Citation 2).

We further selected the novel identified interaction between the proteins NEMO and AMOT; the latter being a regulator of tight junctions and polarity and playing a role in proliferation and migration of cancer cells^20^, but has, so far, not been linked to NF-κB or cell death signaling. We verified their interaction by endogenous co-immunoprecipitation (Fig. 4 and Uncropped Western blots, Data Citation 2), showing the high confidence of PPIs in this dataset.

Moreover, using our iMixPro (intelligent mixing of proteomes) AP-MS strategy for distinguishing true interactors from background proteins^21^, we identified NDP52 (Nuclear domain 10 protein 52 or *CALCOCO2*) and NAP1 (NF-κB activating kinase-associated protein 1 or *AZI2*) as novel interaction partners of the protein A20 (A20 iMixPro AP-MS result and iMixPro ratio histograms, Data Citations 2), validating also these novel identified interactions in our dataset.

## Usage Notes

The obtained protein-protein interaction dataset contains valuable information for further unraveling the molecular mechanisms behind TNFR1-induced NF-κB and cell death signaling, in search for novel targets for pharmaceutical intervention. As mentioned, we lose some interactions that are stored in the BioGRID database and which might thus be true interactions. Therefore, we provide a table with the unprocessed data (Spectral Counts, Data Citation 2), as well as a table including all interactions after removing identifications from control experiments (Interactions filtered by controls, Data Citation 2) and tables with the interactions in the extensive network (Extensive network, Data Citation 2) and the lean network (Lean network, Data Citation 2). The Cytoscape files of the networks are available (Data Citation 2) and allow the user to interactively study the network in more detail.

## Additional information

**How to cite this article**: Van Quickelberghe, E. *et al*. A protein-protein interaction map of the TNF-induced NF-κB signal transduction pathway. *Sci. Data*. 5:180289 doi: 10.1038/sdata.2018.289 (2018).

**Publisher’s note**: Springer Nature remains neutral with regard to jurisdictional claims in published maps and institutional affiliations.

## Supplementary Material
